# The world is not enough – the value of increasing registry data in idiopathic pulmonary fibrosis

**DOI:** 10.1186/s12931-020-01377-1

**Published:** 2020-05-06

**Authors:** C. C. Moor, M. Kreuter, F. Luppi, W. A. Wuyts

**Affiliations:** 1 Department of Respiratory Medicine, Erasmus Medical Center Rotterdam, Rotterdam, the Netherlands; 2 Center for Interstitial and Rare Lung Diseases, Pneumology and Respiratory Critical Care Medicine, Thoraxklinik, University of Heidelberg, Heidelberg, Germany; 3 German Center for Lung Research, Heidelberg, Germany; 4 Respiratory Unit, University of Milano Bicocca. S. Gerardo Hospital, Monza, Italy; 5 Department of Respiratory Medicine, University Hospitals Leuven, Leuven, Belgium

Idiopathic pulmonary fibrosis (IPF) is a rare, progressive interstitial lung disease (ILD) with a poor prognosis [1]. The treatment landscape for IPF has dramatically changed in the last years [2, 3]. In the last decade, phase III trials have shown the efficacy of anti-fibrotic medication on slowing down lung function decline in IPF [4–7]. Since then, post hoc analyses of these trials have tremendously improved our knowledge about the efficacy of anti-fibrotic drugs across a broad spectrum of lung function impairment, the impact of concomitant medication on disease progression, health-related quality of life, and potential prognostic biomarkers [8–14]. Besides, data from open-label extension studies have informed us about the long-term safety, side-effects and tolerability of anti-fibrotic medication [15, 16]. Nevertheless, it should be noted that these clinical trials included a selected subgroup of patients with IPF, with relatively few comorbidities, strict lung function criteria, and age restrictions [4, 5, 17].

During the last years, an increasing number of IPF disease registries have emerged in order to obtain real-world, long-term data in a broader patient population, complementary to clinical trials. In Europe alone, there are over 90 individual IPF registries by now; a minority also collects data about other ILDs [17]. Patients in registries tend to have a slightly worse health-related quality of life, more clinically important comorbidities, and may have a more severely impaired lung function as compared to patients who participated in clinical trials [18]. Real-world registries have proved to be an important source of information regarding disease behavior, use and long-term efficacy of anti-fibrotic medication, mortality, prevalence and impact of comorbidities, burden of disease, and various other topics [19–23]. Despite these advances, many more essential questions remain to be elucidated in the coming years [17].

Holtze and colleagues recently addressed a few of these questions, by publishing a detailed characterization of IPF treatment patterns in the United States (US), using the Pulmonary Fibrosis Foundation Patient Registry (PFF-PR) [24]. The PFF-PR is a multicenter registry with 42 registry sites across the US, which encompasses data about 1224 patients with IPF enrolled between 2016 and 2018 [24]. With their extensive analysis of different factors associated with anti-fibrotic use, the authors have attempted to gain better insights in the selection process on deciding when to start anti-fibrotic treatment and which type. Strengths of this study include the recruitment of a large number of IPF patients with relatively few missing data. This report is among the first in the US to provide real-world data about prescription habits of anti-fibrotic medication. Data of a specific healthcare system could give us additional insights how particular healthcare systems influence treatment issues in IPF.

This study yielded some interesting findings regarding real-world diagnosis and management of IPF in the US [24]. Less than two-thirds (61%) of patients used an anti-fibrotic drug at the moment of enrollment in the registry or in the twelve months before. Not only in the US, but also in many European countries there is still a significant number of patients in which anti-fibrotic therapy is not initiated, despite a confirmed diagnosis of IPF [25]. Moreover, a substantial minority (23%) of patients were using immunosuppressive medication, which is surprising because the detrimental effects of immunosuppression in IPF are well-recognized [26]. Up to one fourth of patients who used anti-fibrotic medication would not have been eligible for clinical trial participation, highlighting the added value of this large amount of real-world registry data. The use of anti-fibrotic medication varied, largely depending on the specific registry site. Unfortunately, a more extensive analysis of factors explaining this variation was hampered by insufficient statistical power. It is likely that diagnosis and treatment patterns are dependent on local experiences and expertise. More research is needed on how to further increase expertise in a rare disease as IPF, which is also revealed by this large US registry. It is clear that in a substantial minority of patients the diagnosis of IPF has been confirmed by an experienced multidisciplinary team (MDT), however, more than half of patients have been diagnosed without validation in an MDT. This is one of the future challenges in the field.

Factors associated with anti-fibrotic use in the PFF-PR registry were recent clinical trial participation, lower diffusion capacity of the lung, oxygen use, and increased time since diagnosis, suggesting that anti-fibrotic drugs are mainly initiated in patients with more severe disease [24]. Data from other registries have shown that the use of anti-fibrotic drugs is associated with prolonged survival, emphasizing the importance of early treatment initiation [23, 27, 28]. However, the substantial heterogeneity of the study population (i.e. recruitment of both incident and prevalent patients), and lack of information about previous medication use, precludes the authors from drawing any definite conclusions about the time from diagnosis to medication initiation. This brings us to the lack of data granularity, which is a potential flaw in the design of the registry. For instance, data about indications for medication use, and information about individual healthcare providers could not be extracted. Consequently, this leads to unfounded speculations, which cannot be accurately verified. Although this may be partly inherent to the use of a real-world registry, it also depends on the selection of outcome measures, and the methods of data collection and quality control.

In this study, Holtze et al. reported on the baseline data of patients enrolled in the PFF-PR registry [24]. Future longitudinal data will likely yield interesting novel information about long-term use, side-effects and efficacy of anti-fibrotic drugs in a real-world US setting. The outcomes of the present study will hopefully help to raise more awareness among US clinicians regarding variations in current clinical practice and prescription patterns, and thereby give directions for future studies and daily care.

Even though this recently published study emphasizes the added value of disease registries in capturing real-life data about diagnosis, treatment and follow-up of IPF patients, it also shows that the use of registry data poses a number of challenges [17, 18, 24, 29]. Due to the real-world data collection, the patient population in registries can be very heterogeneous, and data is often collected both retrospectively and prospectively [18]. Moreover, although selection bias is less overt than in clinical trials, it may still play a role. Most registries recruit patients in ILD expert centers, but not all patients are referred to these centers (e.g. patients with significant comorbidities, elderly patients). The majority of ongoing registry studies are conducted in a single country; only a few European multinational initiatives exist to date [19, 23]. Individual registries have diverse inclusion criteria, the method of data collection and quality control varies, and outcome measures differ. This obviously hampers the interpretation, comparison, and integration of data from multiple registries [17, 18]. For instance, the study by Holtze et al. attempted to compare their findings with other international registries to analyze potential differences in prescriptions patterns across the world; however, due to differences in study design, included outcome measures and enrollment periods, accurate comparisons were unfortunately not possible [24].

A group of ILD experts have recently proposed an idea to create a Europe-wide meta-registry for IPF, consisting of multiple individual registries (ARIANE-IPF) [17]. The availability of a meta-registry will allow for additional (subgroup) analyses and propensity analyses, which generally require large datasets. As also shown by Holtze et al., subgroup analysis is often hampered by the relatively small sample sizes of current registries [24]. Larger datasets may especially be of added value to enhance our knowledge and understanding of rare events, such as acute exacerbations [29, 30]. Finally, a Europe-wide, or even better, global registry could also facilitate analysis of geographical variations in care and outcomes, and differences between healthcare systems, which will hopefully improve equal access to care for patients in the near future [31]. It should however be noted that registries are currently not available in all countries; thus, registry data might still not be completely generalizable. Though the ARIANE-IPF meta-registry initiative yielded much enthusiasm among different stakeholders, some potential issues have been raised too [17]. One of the most challenging issues is the integration of data from different registries. In order to combine individual registries, harmonization of data and standardization of outcome measures is crucial. Unfortunately, the best way to do this has not been fully clarified yet. Furthermore, the number of registries, the amount of data, and the complexity of data that we collect, will probably continue to expand in the coming years. Hence, novel technologies, such as artificial intelligence (AI) applications (e.g. natural language processing, named-entity recognition, and machine learning) could possibly be of added value in the future. For instance, AI has the potential to recognize, extract and recode data from various sources. Moreover, AI may be used for advanced statistical analysis of very large datasets with a wide variety of data (“big data”). Although this may seem far away, AI applications are already studied in registries for other rare diseases [32–34].

Other factors that should be taken into account in the design of registry studies are legal, ethical, privacy, and methodological issues, such as the handling of missing data. Because of the real-world nature, missing data are usually more common in registries than in clinical trials [18]. New technological solutions, such as online home monitoring applications can probably help to minimize the amount of missing data, by increasing patient participation in registries. Patients can be actively involved in the collection of their own data at home, by measuring physiological parameters, and online completion of patient-reported outcome measures about health-related quality of life, symptoms and side-effects. Patients or healthcare providers can even be reminded if data are missing [35]. This would not only enable more frequent data collection, as distances can be bridged online, but would also lower the administrative burden on registry sites.

Ideas about the added value of registry data, current challenges, opportunities, and future directions are summarized in Fig. 1.

**Fig. 1:**
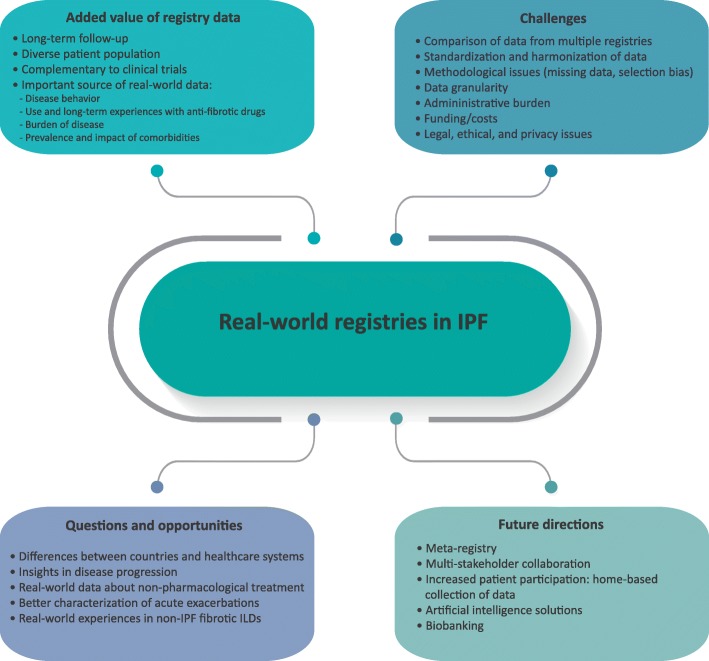
Added value, challenges, opportunities and future directions of real-world registries in IPF

In conclusion, we believe that current challenges can be overcome by increasing multi-stakeholder collaboration in the next years. In that way, registry data will continue to provide answers on clinically relevant questions and improve our knowledge about IPF. Recent phase III trials have also demonstrated the efficacy of anti-fibrotic medication in non-IPF fibrotic ILDs, which will importantly change care for this group of patients [36, 37]. Obviously, real-world data about anti-fibrotic medication use in this patient group is still lacking. The collection of registry data in a more diverse group of fibrotic ILDs, using IPF registries as a model, will be a major opportunity to enlighten factors associated with disease behavior in general, gain much needed insights in disease progression, learn about the clinical use of anti-fibrotic medication including its combination with “standard” therapies, such as immunomodulators, and will facilitate comparison between different diseases.

## Data Availability

Not applicable.

## Abbreviations

IPF: Idiopathic pulmonary fibrosis
ILD: Interstitial lung disease
PFF-PR: Pulmonary Fibrosis Foundation – Patient registry
US: United States
MDT: Multidisciplinary team
AI: Artificial intelligence
